# High prevalence of overall overweight/obesity and abdominal obesity amongst adolescents: An emerging nutritional problem in rural high schools in Limpopo Province, South Africa

**DOI:** 10.4102/phcfm.v13i1.2596

**Published:** 2021-05-18

**Authors:** Sego Debeila, Perpetua Modjadji, Sphiwe Madiba

**Affiliations:** 1 Department of Public Health, School of Health Care Sciences, Sefako Makgatho Health Sciences University, Ga-Rankuwa, South Africa

**Keywords:** overweight and obesity, socio-demography, nutrition knowledge, dietary practices, high school adolescents, rural South Africa

## Abstract

**Background:**

As the gap in nutritional profiles between urban and rural rapidly reduces because of nutrition transition, rural adolescents are likely to engage in urban lifestyle behaviours.

**Aim:**

The study determined the prevalence of overweight/obesity amongst adolescents in rural high schools and the association with selected factors.

**Setting:**

Fetakgomo Municipality in rural Limpopo Province, South Africa.

**Methods:**

A cross-sectional study was conducted amongst 378 adolescents selected through multistage sampling from high schools. Data collected were socio-demography, nutritional knowledge, dietary practices and anthropometry. The International Obesity Task Force age and sex-specific body mass index (BMI) cut-off values were used to determine overweight/obesity, whilst adult BMI cut-off values were used for those ≥ 18 years. Waist-to-height ratio (WHtR) greater than 0.5 indicated abdominal obesity, as well as waist circumference (WC) and waist-to-hip ratio (WHR) above the cut-off values.

**Results:**

The proportion of overweight/obesity amongst adolescents was 35%, whilst 25% had abdominal obesity by WHR and 21% by WHtR. Multivariate logistic regression showed that being a girl (AOR = 2.9, 95% CI: 1.74–4.85), older adolescent (AOR = 3.1, 95% CI: 1.57–6.29) and living in a household with employed adults (AOR = 2.3, 95% CI: 1.19–4.51) were associated with increased odds of being overweight/obese. Eating breakfast was associated with reduced odds of being overweight/obese (AOR = 0.6, 95% CI: 0.34–0.97).

**Conclusion:**

Overweight/obesity and abdominal obesity amongst adolescents were more prevalent than underweight. The Integrated School Health Programme should have clear guidelines on food items served and sold at schools.

## Introduction

Being overweight and obesity are one of the current public health issues^1^ and a challenging problem in developing countries, similar to developed countries.^2^ The World Health Organization (WHO) estimates that 15% of adolescents in Africa are overweight or obese. This is of concern because adolescents represent the largest (1.2 billion) population group in history and 90% of adolescents live in low- and middle-income countries (LMICs).^3,4^ Adolescence is a vulnerable time for the development of obesity because it is marked by a slowing of growth and corresponding decrease in physical activity levels.^5^ Adolescence is a period of growth and development that is increasingly being recognised as a critical window for optimising the health and well-being of current and future generations.^6,7^ Therefore, adolescents who are overweight or obese have an increased risk of metabolic, cardiovascular and other related non-communicable diseases (NCDs), which may persist in adulthood.^8,9^ Therefore, the well-being of adolescents is critical to achieving the sustainable development goals.^10^

The rate of overweight/obesity amongst South African children and adolescents is comparable to rates found more than a decade ago in some developed countries, and is amongst the highest in Africa.^11^ The incidence of overweight and obesity amongst adolescents increased substantially in South Africa between 2002 and 2008, with 11% of boys and 29% of girls between 13 and 18 years of age being overweight or obese in 2008.^12^ In 2013, 19% of boys and 26% of girls < 20 years of age were overweight or obese.^13^ High prevalence of overweight/obesity in adolescents is accompanied by persistent burdens of underweight in most communities, indicating the double burden of malnutrition, which occurs because of nutrition transition.^14,15,16,17^

A shift from traditional diets composed of whole foods to an energy-dense and nutrient-poor diet is defined as nutrition transition.^14^ The literature documents that the increasing prevalence of overweight/obesity is potentially linked to an obesogenic environment, which includes urbanisation, cultural, social and economic issues such as increased wealth and lower levels of physical activity, coupled with high consumption of energy-dense foods.^18,19^ The home environment determines the occurrence of overweight/obesity in children and adolescents.^11^ Monotonous diet mainly based on starches is common in households with food insecurity and contributes significantly to poor dietary practices amongst adolescents.^20^ Majority of adolescents in Africa have poor dietary practices, such as skipping of breakfast and intake of high salt, fat and sugar, as well as lack of physical exercise.^10,21,22,23,24^ Research further indicates that overweight and obesity amongst children and adolescents is strongly dependent on age, gender and populations.^11,25,26^ Addressing these issues in a holistic manner could curb the escalating prevalence of overweight/obesity in the country.

The Strategy for the Prevention and Control of Obesity 2015–2020 has a strong focus on preventing childhood obesity and aims to enable access to healthy food choices in various settings, including schools.^27^ According to the strategy, nutrition education in schools should be in line with national recommendations. The Department of Basic education has developed the manual comprising information on food-based dietary guidelines for teachers who teach life orientation subjects at schools.^28^ In addition, the Integrated School Health Program (ISHP) was introduced in 2012 in South Africa, with the aim of contributing to the improvement of the general health of school-going learners as well as the environmental conditions in the schools’ nutrition education and assessment.^29^ Hence, the dietary patterns of adolescents are mostly shaped by the school environment because they spent a significant amount of time at school. In addition, the school food environment is an important component in effective school-based interventions to promote healthy eating.^30^

However, in general, the school food and nutrition environment are not conducive for promoting healthy eating.^31^ Adolescents consume at least one meal a day at school and the meals either are from the tuck-shop, vendors or from meals provided by the National School Nutrition Program (NSNP), and/or the students’ lunch boxes from home.^30^ The tuck-shops in schools sell snacks and energy dense foods, sweets, sweetened drinks and fat-rich foods. Some students prefer to buy lunch food from the tuck-shops rather than carrying lunchboxes to school^32,33^ and, as a result, consume foods which predispose one to obesity.^34^ It is believed that as nutrition transition advances in rural settings, rural adolescents are likely to uptake urban lifestyle behaviours with poor dietary practices, which have been linked to lack of knowledge about healthy foods amongst adolescents.^22,35^ The ISHP should have clear guidelines on food items served and sold at schools and unhealthy foods should not be tolerated on school premise, as suggested by Okeyo et al.^31^

There is a dearth of recent data on adolescent overweight/obesity in most rural settings of the country. In view of this, the current study aimed to determine the prevalence of overweight/obesity amongst adolescents in rural high schools and the association with socio-demographics, dietary practices and nutritional knowledge. Adolescence is a suitable age for interventions that can enforce good nutritional practices and avert the beginning of NCD that are nutrition-related during adulthood,^10,36,37^ and schools offer an opportune setting for obesity prevention.^38^

## Research methods and design

### Study design, population and setting

This article is part of a dissertation for Masters in Public Health. The study was cross-sectional in design and was conducted in 2017 in the Fetakgomo Municipality, located in Limpopo Province in South Africa. The municipality is rural and made up of a cluster of several villages with similarities in terms of poor infrastructure, economic status and cultural practise. High schools in this area are located across the villages. According to the Education Management Information System (EMIS),^39^ the municipality has four educational circuits consisting of 31 high schools with a total enrolment of approximately 9623 students. The minimum enrolment number in the smallest school is 27 with a maximum of 1004 in the largest school. Most of these schools are situated near shopping complexes with multiple enterprises and small shops established within, including food selling markets where children buy food during lunch breaks and after school. Of note is that according to reports from Statistics South Africa, about 87% of learners in rural areas in Limpopo Province walk to school and, at most, take about half an hour to reach school.^40^

### Sample size and sampling technique

A sample size was calculated using Rao software size calculator.^41^ A population of 9623 (5% margin of error, 95% confidence interval [CI]) was used to calculate a minimum sample of 370 participants. A multistage sampling technique was used to select schools and learners. First, the high schools were stratified by the size of enrolment and four largest schools were selected. Learners were recruited through class teachers and consent forms were distributed to seek permission to participate in the study from their parents. Taking note of parental consent, we grouped participants by their grades and the participating students were randomly selected from each grade. Learners without parental consent and below 18 years were excluded during sampling, as well as those who had a disability that compromised stature. Each selected school was treated as a unit of analysis with a sample size of 95 learners to avoid disproportionate sampling amongst the four selected school, and a final sample of 380 high school adolescents was obtained. Adolescence is the transitional stage from childhood to adulthood that occurs between ages 13 and 19.^42^ The stages of adolescence include early adolescence from age 10 to 14, mid-adolescence from age 15 to 17 and late adolescence from age 18 to 24. Each stage was represented.^42^ We considered all learners who were attending high schools at the time of the study.

### Data collection

Information on socio-demography, dietary practices and nutrition knowledge was collected from the adolescents, and anthropometry was measured. Prior to data collection, a written consent was obtained from the parents whose children participated in the study.

### Socio-demography

A validated questionnaire was distributed to learners to fill information on the socio-demographic factors. The questionnaire covered a range of socio-demographic characteristics and the household situation of adolescents, in accordance with the variables used in other studies conducted in rural areas of Limpopo Province.^43^ Personal information collected entailed age, gender, home language, grade and religion. Household information included household size, employment status of adults, income, type and size of the house the participant lived in; availability and sources of water and energy, type of sanitation and refrigerator use.

### Nutrition knowledge and dietary practices

Nutrition knowledge was measured using a set of 10 true or false questions, adapted from a study by Kigaru et al.^22^ For each question, a correct response was coded as ‘1’ and an incorrect response as ‘0’. The total score for every adolescent was calculated from all correct responses with a maximum of 10. This was then converted to a percentage and the scores for nutrition knowledge were categorised as low (≤ 40%), moderate (41% – 69%) and high (≥ 70%). The dietary practices were determined using a validated food frequency questionnaire (FFQ) with foods arranged in nine categories based on food grouping.^22^ A list of commonly available soft drinks and fast foods was provided for the adolescents to indicate the frequency of consumption in the last 7 days prior to the study to compute the total frequency of consumption. The consumption of foods more than four times in a week was considered excess consumption, whilst consumption of less than four times in a week was considered deficient intake.

### Anthropometric measurements

Weight and height of adolescents were measured using smart D-quip electronic scale and stadiometer, respectively, based on the WHO standard procedures.^44^ Body mass index (BMI) was categorised using the International Obesity Task Force (IOTF) classifications. For adolescents aged below 18 years, the absolute age and sex-specific cut-offs for BMI were used, defined as a BMI of 25 kilograms per square metre (kg/m^2^) and 30 kg/m^2^ for overweight and obesity, respectively. For those aged 18–20 years, adult cut-off points of a BMI ≥ 25 kg/m^2^ and ≥ 30 kg/m^2^ for overweight and obesity were used. Waist circumference (WC) and hip circumferences (HC) were measured using a non-stretchable plastic tape measure. Central obesity was defined as WC ≥ 94 centimetres (cm) for males and ≥ 80 cm for females.^44,45^ Abdominal obesity was defined at waist-to-hip ratio (WHR) more than 0.90 for males and 0.85 for females whilst waist-to-height ratio (WHtR) of 0.5 was used for both sexes.^46^

### Data analysis

STATA was used to analyse data. Data were presented as frequencies and percentages (i.e. categorical variables) and means and standard deviations (s.d.) (i.e. continuous variables). Chi-squared test compared the percentages, whilst the Student’s *t*-test compared the means. The association of overweight/obesity with socio-demographic characteristics, nutritional knowledge and dietary practices was computed using a multivariate logistic regression analysis. At bivariate analysis, independent variables associated with overweight/obesity at a *p* ≤ 0.2 were included in the multiple logistic regression analysis, using forward stepwise regression. Significance was set at *p* < 0.05.

### Ethical considerations

This study was conducted according to the guidelines laid down in the Declaration of Helsinki^47^ and all procedures involving human subjects were approved by Sefako Makgatho Health Sciences University Research and Ethics Committee (SMUREC/H/42/2017:PG). Furthermore, this study received permission from the Provincial Department of Education, South Africa. Written informed consent was obtained from parents of adolescents younger than 18 years and written assent from older learners. Adolescents 18 years and older signed their own consent forms.

## Results

### The characteristics of adolescents

The demographics of the 378 high school adolescents are presented in Table 1. The majority (62%) were females, 76% were < 18 years and 24% were ≥ 18 years. The mean age for adolescents was 16 years (s.d. = ±2), and the mean age of 16 years (s.d. = ±2) was similar in boys and girls (*p* = 0.111). Most adolescents lived in brick houses (85%), and the majority (97%) used electricity. Although 96% had access to municipal water, pit toilets were commonly used (87%). Over two-thirds (63%) lived in households that were large (≥ 5 members), with a monthly income of less than $183.01 (30%), whilst 49% did not know their household income, and 22% came from households with no employed adults.

**TABLE 1:** Demographic characteristics of adolescents.

Variables	Categories	Frequency (*n*)	Percentages
Age (years)	13–17	286	76
	18–20	92	24
Gender	Boys	143	38
	Girls	235	62
Level of study	Grade 8	65	17
	Grade 9	91	24
	Grade 10	43	11
	Grade 11	97	26
	Grade 12	82	22
House type	Brick	323	85
	Non-brick	55	15
Household size	< 5 members	138	37
	≥ 5 members	240	63
Household income	≤ $61.01	55	15
	$61.01–$183.01	55	15
	$183.01–$305.02	24	6
	$305.02–$610.05	30	8
	≥ $610.05	27	7
	Don’t know	187	49
Number of employed adults	0	83	22
	1	168	44
	> 1	127	34
Toilet	Pit	329	87
	Flush	49	13
Electricity	No	12	3
	Yes	366	97
Fridge	No	50	8
	Yes	348	92
Water	Municipal water	361	96
	River	17	4

### Anthropometric characteristics of adolescents

Table 2 presents BMI status of the adolescents. Overall overweight/obesity was 35%, with 26% and 9% of adolescents being overweight and obese, respectively, whilst 6% were underweight. More of the girls (32%) than boys (16%) were overweight; more girls (11%) than boys (6%) were obese, whilst more boys (10%) than girls (4%) were underweight (*p* ≤ 0.0001). Central or abdominal obesity was significantly different by gender: girls had higher prevalence of overweight/obesity (43%) compared with boys (21%), and abdominal obesity (by WC; 13% vs. 2% and WHR; 30% vs. 17%). The observed differences were statistically significant (*p* ≤ 0.0001). Overweight and obesity were merged as one variable for the analysis.

**TABLE 2:** Anthropometry of adolescents in high schools by gender.

Variables	All		Boys		Girls		*P*
	*n* (378)	%	*n* (143)	%	*n* (235)	%	
Normal BMI	221	59	97	68	124	53	≤ 0.0001^*^
Underweight	24	6	14	10	10	4	
Overweight	99	26	23	16	76	32	
Obesity	34	9	9	6	25	11	
Normal weight	222	59	98	69	124	53	≤ 0.0001^*^
Underweight	24	6	14	10	10	4	
Overweight/obesity	132	35	30	21	102	43	
Normal WC	334	91	140	98	204	87	≤ 0.0001^*^
Abdominal obesity	34	9	3	2	31	13	
Normal WHR	283	75	119	83	164	70	0.004^*^
Abdominal obesity	95	25	24	17	71	30	
Normal WHtR	297	79	116	81	181	67	0.346
Abdominal obesity	81	21	27	19	54	23	

BMI, body mass index; WC, waist circumference; WHR, waist-to-hip ratio; WHtR, waist-to-height ratio.

* , Significant differences, BMI: normal (18.5 kg/m^2^ – 24.9 kg/m^2^), underweight (< 18.5 kg/m^2^), overweight (25 kg/m^2^ – 29.9 kg/m^2^), obesity (≥ 30 kg/m^2^); WC: normal (< 88 cm), abdominal obesity: ≥ 94 cm for males and ≥ 80 cm for females; WHR: normal (< 0.85), abdominal obesity: > 0.90 for males and 0.85 for females; WHtR: normal (< 0.5), abdominal obesity (≥ 0.5).

### Nutrition knowledge of adolescents

Table 3 shows the correct responses of the adolescents on nutrition knowledge, based on the literature.^22,48,49^ Two-thirds (64%) knew that the mid-day meal is not as important as breakfast, 81% knew that it is necessary to drink 2 litres (L) of water a day. A minimum daily fluid requirement of 2 L per day has been acknowledged.^48^ Eighty-four per cent of participants understood the need to exercise, whilst 79% knew that eating too much meat was not good. Too much consumption of meat is defined as more than three portions per week.^49^ Seventy-five per cent of participants knew that consumption of fruits and vegetables daily is necessary. Most (81%) knew that eating too much sugar was not good, 65% knew that drinking juice is not as healthy as eating a portion of fruit, and 34% did not agree with the statement that one should drink at least three cups of full cream milk every day. The majority (70%) had high knowledge, 20% had moderate knowledge and 10% had low knowledge (Table 4). The mean knowledge score was 7.2 (s.d. = ±1.9), with a minimum point of 1 and maximum points of 10. The median score was 9, with the lower quartile at eight and the upper quartile at 10. This indicates that the level of knowledge amongst most adolescents was high.

**TABLE 3:** Proportions of adolescents with correct scores in various nutrition knowledge aspects.

Nutrition knowledge aspect tested	Number	% correct answers
Lunch is a more important meal than breakfast	243	64
Boiled eggs are a better option than fried eggs	297	79
You can eat as much meat as you want every day	300	79
It is necessary to eat fruits and vegetables every day	284	75
Potato chips are a healthy way to eat potato	318	84
If you eat healthy food, there is no need to exercise	317	84
Drinking juice is as healthy as eating a piece of fruit	246	65
Eating a lot of sugar gives enough energy	307	81
Drinking three cups of milk every day is important	127	34
It is necessary to drink two litres of water a day	301	80
**Nutrition knowledge**		
Low	38	10
Moderate	76	20
High	264	70

**TABLE 4:** Practices and frequency of consumption of various food items in the last 7 days.

Consumption/frequency	Characteristics	*N*	%
Number of meals consumed per day	One	9	2
	Two	56	15
	Three	313	83
Eat breakfast every day	Yes	279	74
	No	99	26
Eat lunch every day	Yes	250	66
	No	128	34
Eat supper every day	Yes	293	78
	No	85	22
Commonly consumed breakfast foods	Bread	130	34
	Cereal	119	32
	Porridge	79	21
	Any food available	50	13
Lunch foods consumed	Food from school	274	73
	Foods bought from tuck-shop	77	20
	Lunch box	27	7
Common foods bought from tuck shop	Fries at least once a week	248	66
	S’phatlho^†^ at least once a week	357	94
	Sweetened drink every day	173	46
Commonly consumes supper foods	Pap and spinach 2–3 times a week	81	21
	Pap and eggs 2–3 times a week	22	6
	Pap and non-meat relish 2–3 times a week	49	12
	Pap and meat at least once a week	226	60
Eat fruits once a week	Yes	104	28
	No	274	72
Eat vegetables 2–3 times a week	Yes	135	36
	No	243	64
Sleep hungry once a week	Yes	65	18
	No	312	82

† , Quarter-loaf of white bread filled with chips, a slice of cheese, atchaar and delicatessen meats and sauces.

### Dietary practices of adolescents

Table 4 shows the dietary habits and eating practices in the last 7-day period. Most (83%) consumed three meals per day and 18% went to sleep hungry at least once in a week. The majority (74%) ate breakfast, 66% ate lunch and 78% ate supper every day. Common breakfast foods consumed were bread (34%), cereal (32%) and porridge (21%). During lunchtime, 73% ate food provided by the school, 20% bought food from the tuck-shops and 7% brought lunch boxes. Pap and non-meat relish (40%) were often consumed as a supper meal rather than pap and meat that were consumed once in a week (60%). Sweetened drinks (42%) were consumed daily, whilst low fruit (28%) and vegetable (36%) consumption was observed per week.

### Factors associated with overweight/obesity

The determinants of overweight/obesity are shown in Table 5. In the bivariate logistic regression analysis, ages (17–18 years; *p* = 0.005) and (19–20 years; *p* = 0.014), gender (*p* ≤ 0.0001), and the number of household adults employed (one member; *p* = 0.020) and (> one member; *p* = 0.018) were associated with overweight/obesity. Furthermore, bivariate analysis showed that girls were significantly affected by abdominal obesity compared to boys (WHR; OR = 2.7, 95% CI: 1.23–3.49) and (WC; OR = 7.0, 95% CI: 2.10–23.37). No significant association was observed between overweight/obese and nutritional knowledge at the bivariate level.

**TABLE 5:** Logistic regression analysis of correlates for overweight/obesity amongst adolescents.

Overweight/obesity	Crude		*p*	Adjusted		*p*
	OR	95% CI		OR	95% CI	
**Age category (years)**						
13–14	-	-	-	1	-	-
15–16	1.6	0.9–3	0.147	1.9	0.98–3.69	0.058
17–18	2.6	1.3–5.1	0.005*	3.0	1.45–6.08	0.002**
19–20	2.3	1.2–4.3	0.014*	3.1	1.57–6.29	0.001**
**Gender**						
Boys	-	-		1	-	-
Girls	2.6	1.6–4.2	≤ 0.0001*	2.9	1.74–4.85	≤ 0.0001**
**Household adults employed**						
0	-	-	-	1	-	-
1	2.0	1.1–3.7	0.020*	2.0	1.05–3.75	0.035**
> 1	2.1	1.1–3.9	0.018*	2.3	1.19–4.51	0.013**
**Eating breakfast**						
No	-	-	-	1	-	-
Yes	0.5	0.3–0.9	0.023*	0.6	0.34–0.97	0.040**

Note: 1 indicates reference.

OR, odds ratio; CI, confidence interval.

* , Significant difference at bivariate level.

** , significant difference at multivariate level.

At multiple logistic analyses, overweight/obesity was significantly associated with age, gender, number of adults employed in the household and eating breakfast. The odds of being overweight/obese was 1.9 times for adolescents aged 15–16 years (adjusted odds ratio [AOR] = 1.9, 95% CI; 0.98–3.69), three times for those 17–18 years (AOR = 3.0, 95% CI; 1.45–6.08), and 3.1 times for 19–20-year-olds (AOR = 3.1, 95% CI; 1.57–6.29). Girls were more likely to be overweight/obese compared with boys (AOR = 2.9, 95% CI: 1.74–4.85). Adolescents living in households with at least one employed adult (AOR = 2.0, 95% CI: 1.05–3.75) and more than one employed adult (AOR = 2.3, 95% CI: 1.19–4.51) were more likely to be overweight/obese than those living in households with no employed adult. Eating breakfast reduced the odds of being overweight/obese (AOR = 0.6, 95% CI: 0.34–0.97).

## Discussion

The aim of this study was to determine overweight/obesity prevalence, and further study its associations with socio-demography, nutritional knowledge and dietary practices of adolescents in high schools in Limpopo Province, South Africa. The findings showed a high prevalence of overweight/obesity amongst adolescents, similar to several studies in South Africa and sub-Saharan Africa (SSA).^11,50,51,52,53^

The high overall prevalence of overweight/obesity (35%) far outweighed those who were underweight (6%) amongst adolescents in this poorly resourced community. Over a third (36%) lived in households where the income of employed adults ranged from $55.00 to $264.00 per month. Nevertheless, overweight/obesity was prevalent in households with at least one employed adult than those with no employed adults. Yet, the diet of adolescents was an energy-dense and nutrient-poor diet mainly based on starches. In a recent review, Wrottesley et al.^34^ suggested that diets that lack diversity, originating from poverty, are persistent in some rural communities in South Africa. The link between poor nutritional intake and overweight/obesity amongst adolescent girls has been well established.^10^

The overall overweight/obesity prevalence in this study is higher than the national range of 8.6% – 27.0% amongst adolescents aged 15–19 years reported by the United Nations Children’s Fund.^54^ Similarly, the prevalence of overall overweight/obesity is higher than in other studies in urban and rural settings in South Africa. A recent study by Negash et al.^53^ reported overweight/obesity prevalence of 22.9% amongst adolescents in urban high schools in more affluent communities in the Western Cape in South Africa. The overweight/obesity rate reported in this study is higher than values reported in earlier studies conducted in South Africa,^21,53,55,56^ and rates reported in urban schools in Ghana, Ethiopia and Nigeria.^50,51,52,57^ Our results are similar with worldwide implications with the difference that obesity in the rural and the urban is decreasing, and the rate of overweight/obesity is increasing amongst populations in the rural setting.^14^

Our results showed a significantly higher overweight/obesity amongst in girls (43%) than in boys (21%). The difference between rates reported in boys and girls is comparable with rates reported in several studies in South Africa,^21,53,55,56^ rates in SSA^50,51,52^ and rates in national estimates.^26,53^ However, the prevalence reported in girls (43%) is higher than the 26.3% national prevalence reported in South Africa in 2013.^13^ Research has shown that girls naturally require less energy intake than boys on a biological level.^58^ On a behavioural level, girls are reported to be more attentive to food and its effects on health and weight control.^59^ In addition, adolescent girls are less likely to meet physical activity recommendations than boys.^60^ Other studies, mostly conducted in urban schools in South Africa, reported lower prevalence of overweight/obesity in girls^21,53,56^ as well as studies conducted in rural schools.^61,62^ The prevalence reported in this study is higher than values reported in urban schools in Ghana, Ethiopia and Nigeria.^50,51,52,57^

In addition to the high overall overweight/obesity prevalence, the results found a high prevalence of abdominal obesity indicated by elevated WHtR (21%), elevated WHR (25%) and elevated WC (9%). Girls were more significantly affected by abdominal obesity (by WC and WHR) compared with boys. Similarly, several studies have reported abdominal obesity (by WC, WHR and WHtR) amongst adolescents in South Africa.^63^ The finding reported in the current study is consistent with those reported amongst students in Ghana, which highlighted higher odds of abdominal obesity amongst females compared to males.^36,64^ Adolescent females were almost twice as likely to be abdominally obese than males based on the WHtR and WHR indicators. The abdominal obesity rates reported in the current study has significant public health implications. Compared with generalised obesity, abdominal obesity in children and adolescents is more strongly correlated with metabolic risk factors.^65,66^ Of public health importance is the link between the onsets of abdominal obesity with modifiable lifestyle patterns;^36^ this suggests that obesity prevention interventions should be context and culturally appropriate.

An increase in overweight/obesity rate with age has been reported in studies and national estimates in SSA.^61^ This study showed that the odds of being overweight/obese increased with age; adolescents aged 18–20 years were more likely to be overweight or obese compared to those below 18 years. The finding is parallel with a 20-year follow up South African study which found that overweigh/obesity increased throughout childhood, whilst the trend in overweight/obesity amongst girls followed the onset of puberty.^67^ The study found that the prevalence increased in the periods from 15 to 16 years to 17 to 18 years and from 19 to 20 years. The 2012 South Africa National Health and Nutrition Examination Survey revealed that the adults who are obese was consequent to late adolescence.^68^ Preventing overweight and obesity amongst adolescents is a public health approach to prevent obesity amongst adults.^50^ Therefore, the data underscored the need to identify modifiable risk factors such as television viewing time for long periods to develop interventions to reduce the occurrence of overweight and obesity.^67^

Although no association was observed between dietary patterns and overweight/obesity amongst adolescents by other researchers,^35^ the current study found significant association between skipping breakfast and overweight/obesity. Adolescents who reported to consume breakfast had 40% reduced odds of being overweight or obese. This is consistent with a Nigerian study amongst learners in urban schools.^23^ In line with several studies, 26% adolescents frequently skipped breakfast.^33,38,69^ A recent review of South African studies found that food unavailability and inaccessibility to healthy food in some households deprive the consumption of breakfast amongst learners in rural settings.^34^ Skipping breakfast might lead overeating during other meal-times, compromise healthy eating, which could induce overweight/obesity.^34,69^ The link between eating breakfast and low snacking is reported in several studies.^66,70,71^ Appropriate and context-relevant interventions to promote healthy dietary practices are crucial to stem the tide of adolescent overweight/obesity.

## Limitations

It is important to interpret the findings of the current study with an understanding because a cross-sectional descriptive study was used. We only used food frequency to recall the types and frequency of foods consumed, but did not quantify the energy and nutrients of foods consumed by adolescents. Nonetheless, we were able to study the dietary practices of adolescents and learn about the kind of foods they access with ease in the school environment. There is a possibility that the absence of a significant association between overweight/obesity and dietary intake might be influenced by the incorrectness to in dietary measurements, as well as dietary recall, and under-reporting of other foods such as rich-carbohydrates foods usually consumed by the obese individuals.

## Conclusion

The study reported a high prevalence of overweight/obesity amongst adolescents in these poorly resourced communities. The reported prevalence was higher than the rates reported in the national estimates and the rates reported in urban studies. Overweight/obesity was more prevalent than underweight amongst adolescents, especially amongst girls, and increased with age. There is a need to bring up children and adolescents in a health-promoting environment in an effort to reverse and stop the increased trend of overweight and obesity. Therefore, it is crucial that interventions address the occurrence and management of overweight and obesity in adolescents, seeing as adolescence obesity may persist into adulthood. Multi-stakeholder interventions focus on improving nutritional knowledge of children and adolescents to enable them to make healthier food choices and undertake dietary practices like eating breakfast, amidst existing programmes. Moreover, modifiable household vulnerabilities such as a poor socio-economic status should be considered in the intervention programs.

In addition, the school is viewed as an ideal health-promoting environment that can develop children and adolescents physically, emotionally and socially. The Department of Basic Education, South Africa, sees the school as a place to feed and educate learners but mostly to address undernutrition.^72^ However, despite the fact that school nutrition programmes have many advantages, with reference to the South African context, the service has been beset by management challenges.^73^ The ISHP should have clear guidelines on food items served and sold at schools and unhealthy foods should not be tolerated on school premises, as suggested by Okeyo et al.^31^ Addressing these issues in a holistic manner could curb the escalating prevalence of overweight/obesity in the country.
